# Single time point quantitation of cerebral glucose metabolism by FDG-PET without arterial sampling

**DOI:** 10.1186/s13550-023-01049-3

**Published:** 2023-11-30

**Authors:** Paul Cumming, André H. Dias, Lars C. Gormsen, Allan K. Hansen, Ian Alberts, Axel Rominger, Ole L. Munk, Hasan Sari

**Affiliations:** 1 Department of Nuclear Medicine, Bern University Hospital, Freiburgstrasse 18, INO B 214.C, 3010 Bern, Switzerland; 2 School of Psychology and Counselling, Queensland University of Technology, Brisbane, Australia; 3 Department of Nuclear Medicine and PET Centre, Aarhus University Hospital, Aarhus, Denmark; 4 Department of Clinical Medicine, Aarhus University, Aarhus, Denmark; 5 Advanced Clinical Imaging Technology, Siemens Healthcare AG, Lausanne, Switzerland

**Keywords:** PET, FDG, Brain, Kinetics, Image-derived input function

## Abstract

**Background:**

Until recently, quantitation of the net influx of 2-[^18^F]fluorodeoxyglucose (FDG) to brain (*K*_i_) and the cerebrometabolic rate for glucose (CMR_glc_) required serial arterial blood sampling in conjunction with dynamic positron emission tomography (PET) recordings. Recent technical innovations enable the identification of an image-derived input function (IDIF) from vascular structures, but are frequently still encumbered by the need for interrupted sequences or prolonged recordings that are seldom available outside of a research setting. In this study, we tested simplified methods for quantitation of FDG-*K*_i_ by linear graphic analysis relative to the descending aorta IDIF in oncology patients examined using a Biograph Vision 600 PET/CT with continuous bed motion (Aarhus) or using a recently installed Biograph Vision Quadra long-axial field-of-view (FOV) scanner (Bern).

**Results:**

Correlation analysis of the coefficients of a tri-exponential decomposition of the IDIFs measured during 67 min revealed strong relationships among the total area under the curve (AUC), the terminal normalized arterial integral (theta_(52–67 min)_), and the terminal image-derived arterial FDG concentration (Ca_(52–67 min)_). These relationships enabled estimation of the missing AUC from late recordings of the IDIF, from which we then calculated FDG-*K*_i_ in brain by two-point linear graphic analysis using a population mean ordinate intercept and the single late frame. Furthermore, certain aspects of the IDIF data from Aarhus showed a marked age-dependence, which was not hitherto reported for the case of FDG pharmacokinetics.

**Conclusions:**

The observed interrelationships between pharmacokinetic parameters in the IDIF measured during the PET recording support quantitation of FDG-*K*_i_ in brain using a single averaged frame from the interval 52–67 min post-injection, with minimal error relative to calculation from the complete dynamic sequences.

## Introduction

The quantitation and compartmental analysis of the cerebral uptake and trapping of tracers for positron emission tomography (PET) is frequently hindered by the need for a continuous arterial input function. Serial arterial sampling was a feature of the first phase of PET studies with the glucose analog 2-[^18^F]fluorodeoxyglucose (FDG) [1–3]. While that approach afforded estimation of the individual microparameters in the compartmental model for cerebral uptake and trapping of FDG, serial arterial blood sampling is an invasive procedure that is logistically challenging to perform. In this regard, venous sampling is less problematic, but introduces error due to arterial-venous differences in the FDG concentration, which can be partially overcome by limb-warming procedures aiming to provide “arterialized venous blood” [4], or by scaling of a population-based input function to a single arterialized venous blood sample [5]. Alternately, the plasma integral and convolution in the operational equation for FDG uptake can be estimated from two measured blood samples [6]. In yet another approach, a population based arterial input function gave excellent agreement with IDIF estimates of the net cerebral uptake of FDG relative to an individually measured input [7].

Despite generally good agreement of such approaches with evaluation of FDG uptake relative to serial arterial blood samples, their use has not become widespread. This may reflect in part the general acceptance of global normalization procedures for group comparisons of brain FDG uptake [8, 9]. Indeed, we [10] and others [11] have seen good correlation between static (SUV) and dynamic (*K*_i_; ml g^−1^ min^−1^) quantitation of FDG uptake in brain and other organs, which might call into question the need for absolute quantitation of FDG-PET in most applications. However, we note that global normalization procedures in brain studies can lead to misinterpretation of findings in neurodegenerative diseases [12, 13], and thus we contend that physiologically defined parameters such as *K*_i_ can be more relevant for the assessment of brain metabolism in health and disease conditions. Image-derived arterial input functions (IDIF) are well-established for quantitation of *K*_i_/CMR_glc_ in experimental animals, based on observations of the blood pool in the heart ventricle (i.e., [14, 15]), with spill-over correction of the heart signal [16]. The near absence of plasma metabolites of FDG validates the use of an IDIF as a surrogate measure of the arterial input curve, since nearly the entire radioactivity in the blood compartment is untransformed FDG. Here, the quantitative approach is favored when the organ of interest (brain) and the heart fall within the field of view of the small animal PET.

In human PET recordings with limited field of view, repeated bed motion to record in alternation from the heart and head can enable noninvasive quantitation of FDG-*K*_i_ [17]. For example, good quantitation was obtained in clinical pediatric research by a hybrid approach in which the IDIF was measured from the left ventricle during the initial 17 min of FDG circulation, followed by venous blood sampling during the later phase with head recording [11]. In Aarhus, we have obtained accurate and unbiased estimation of CMR_glc_ in adults using a late phase head recording (50–70 min post injection) combined with a population-based continuous IDIF (0–70 min) obtained through bed motion in the Biograph Vision 600 PET/CT (Siemens Healthineers) scanner [18]. As an alternate to this two-phase scanning approach, the requirement for bed motion in human PET studies might be avoided by obtaining an IDIF from the carotid artery, which falls within the field of view of contemporary PET scanners. However, the small diameter of the lumen of the human carotid (6 mm; [19]) is of the same scale as the full width at half maximum (FWHM) resolution of positron emission tomographs, which calls for measures aiming to correct for spillover or signal loss, i.e., partial volume correction [20]. In Bern, we have used the first clinically installed large field of view (LAFOV) PET-system (Bern; Biograph Vision Quadra (Siemens Healthineers)) to obtain continuous aortic IDIF and whole body recordings for quantitation of the FDG uptake in brain and peripheral tissues, and likewise tested a population-based IDIF in conjunction with an abbreviated scanning protocol [21]. We and others have employed a scaled population-based IDIF to obtain good quantitation of *K*_i_ using an attenuated scanning protocol [22–24].

These diverse approaches address the general need for simplified and reliable methods for quantitation of FDG PET, ideally without any need for interrupted or continuous PET recordings. In the present study, we undertook to develop abbreviated methods for the absolute quantitation of cerebral FDG uptake, relative to IDIFs obtained from the descending aorta as imaged by multiple passes of bed motion (Aarhus; Biograph Vision 600 PET/CT), or with single bed whole body imaging (Bern; Biograph Vision Quadra PET/CT). We first undertook linear graphic analysis of FDG uptake in whole gray matter (GM) and whole white matter (WM) using the entire 67 min dynamic recordings in groups of oncology patients examined in Aarhus or Bern. Due to imperfect harmonization of the imaging protocols at the two sites, we did not pool the data in our analysis, but rather compared the platform stability of our approach. Next, we undertook a pharmacokinetic analysis of the individual IDIFs to identify features that could be used to capture the plasma integral (area under curve; AUC) knowing either the late phase of the IDIF (35–67 min post injection) or only the terminal blood FDG concentration (Ca(_52–67 min_), with a single PET frame obtained by averaging the frames recorded in the interval 52–67 min post FDG injection. We constrained our two point linear graphical analyses (*K*_i_; ml g^−1^ min^−1^) by the extrapolated V_D_ at time zero, as described below.

## Methods

### Aarhus data

The Aarhus study was a retrospective evaluation of prospectively collected clinical FDG PET/CT data, approved by the local ethics committee of the Central Denmark Region (1-10-72-188-19). Inclusion into the dynamic whole-body (D-WB) protocol was solely based on whether patients were deemed fit to lie still for nearly 70 min while in the PET/CT scanner; in practice, this excluded patients with cognitive impairments. The study population of 52 (28 F, 24 M) patients had been referred for FDG-PET due to infection/inflammation (*n* = 11), lymphoma (*n* = 14), lung cancer (*n* = 7), and various other oncological indications (*n* = 30) [25].

Aarhus participants were scanned using a fully automated multiparametric PET acquisition protocol (Multiparametric PET Suite AI, Siemens Healthineers, Knoxville, TN, USA) on a Siemens Biograph Vision 600 PET/CT scanner (Siemens Healthineers, Knoxville, TN, USA) with 26.2 cm axial field of view. First, we performed a low-dose whole body CT (25 Ref mAs, 120 kV, CareDose4D, CarekV, Admire level 3), and then initiated PET recording at the start of a standardized injection of FDG (4 MBq/kg) using an Intego PET Infusion System (MEDRAD, Inc., Warrendale, PA, USA). The 70-min PET protocol consisted of an initial 6-min dynamic scan of the chest region, including the aorta, heart and liver, followed by a 64-min dynamic WB-PET, which consisted of 16 continuous bed motion passes: 7 × 2-min WB passes followed by 9 × 5-min WB passes. PET images were reconstructed using software version VG76A and list-mode data extending to approximately 67 min post-injection (reconstruction parameters: TrueX + TOF, 4 iterations, 5 subsets, 440 matrices, voxel size 1.65 × 1.65 × 1.65 mm^3^, 2-mm Gaussian filter and relative scatter correction). SUV images were normalized to body weight and injected dose.

After acquisition, the automated scan protocol automatically identifies anatomical structures on the low-dose WB CT scan [26], and places a 1.6 mm^3^ cylindrical VOI in the proximal descending aorta [15]. The aorta-VOI was inspected for motion, and an IDIF extracted from the full dynamic PET series of the chest region (0–70 min). The aorta IDIF was recorded at intervals of 0.1 min for the first minute post injection, 0.5 min for the next five minutes, two minutes for the next 15 min, and at five minute intervals, with frame truncation at 67 min post injection, for harmonization of scan duration with the Bern data.

We used the SUV head image for GM and WM matter VOI definition in Aarhus. Each subject's skull and brain was manually isolated using a box-shaped VOI, with rigid matching of the isolated head to an FDG PET template included within PMOD. Tissue probability maps of GM, WM, and cerebrospinal fluid of each subject were then generated using PMOD's adaption of the unified segmentation method from the SPM8 or SPM12 toolbox (https://www.fil.ion.ucl.ac.uk/spm/). Although the tool “MRI Probability/Inhomogeneity” was developed for MRI images, it performs reasonably well for FDG PET data in conjunction with carefully chosen cut-off values. Individual VOIs were generated using a cut-off of > 99.5% for GM probability and > 90% for WM, based on pilot evaluation of the first few subjects, with quality control according to visual inspection of the overlay maps.

### Bern data

The Bern data consisted of FDG-PET scans from 24 patients (9 women, 15 men) under investigation for lymphoma (*n* = 9), breast cancer (*n* = 6), lung cancer (*n* = 4) and various other tumor types (*n* = 5). Details of the analysis of data from these patients are reported elsewhere [21]. The local Institutional Review Board approved the study (KEK 2019–02193), and all patients provided written informed consent. FDG was administered as a single intravenous bolus at a radiochemical dose of 3 MBq/kg. PET data were acquired on a Siemens Biograph Vision Quadra [19]. Acquisition of list-mode PET data started 15 s before the intravenous bolus injection. The emission data were binned into 62 frames of the following durations: 2 × 10 s, 30 × 2 s, 4 × 10 s, 8 × 30 s, 4 × 60 s, 5 × 120 s, and 10× 300 s, with linear truncation of the final frame at 67 min for harmonization with Aarhus data. Frame-wise images were reconstructed using a proprietary software prototype for image reconstruction (e7-tools version VR10, Siemens Healthineers) employing the PSF + TOF algorithm (four iterations and five subsets). The final images were reconstructed in voxels measuring 1.65 × 1.65 × 1.65 mm^3^, with smoothing using a post-reconstruction Gaussian filter (FWHM 2 mm). Images were reconstructed with the high sensitivity mode, which employs a maximum ring difference of 85. The PET data were corrected for randoms, scatter, attenuation, and radioactive decay, following the methods used as clinical standard. Standard uptake value (SUV) images were generated by normalizing the late frame images to body weight and injected dose. Attenuation correction of PET emission data was by means of co-registered low-dose CT scans (voltage: 120 kV, tube current 25 mA, CareDose4D, CarekV).

Segmentations of the aorta were obtained using a deep-learning based method employing spatial ensemble learning [27]. The automatically-obtained aorta segmentations were manually edited to isolate the descending aorta (10 mm luminal diameter cylinders) in which the IDIF was computed as described previously [21]. The IDIF was recorded at intervals of 0.05 min for the first 1.5 min post injection, every 0.25 min for the next 3.5 min, at one-minute intervals for the next 12.5 min, and every five minutes thereafter until the end of the PET recording. We extracted brain GM and WM VOIs using the FDG healthy brain template in standard-space (available in PMOD v.4.1, PMOD Technologies, Zurich, Switzerland). This processing step entailed cropping and spatial normalization of each subject’s brain SUV image to the template using rigid and non-rigid registration methods [28]. Next, we applied the atlas-defined GM and WM templates to the subject’s native space PET images, with manual inspection of the registration.

### IDIF kinetics

**Fig. 1:**
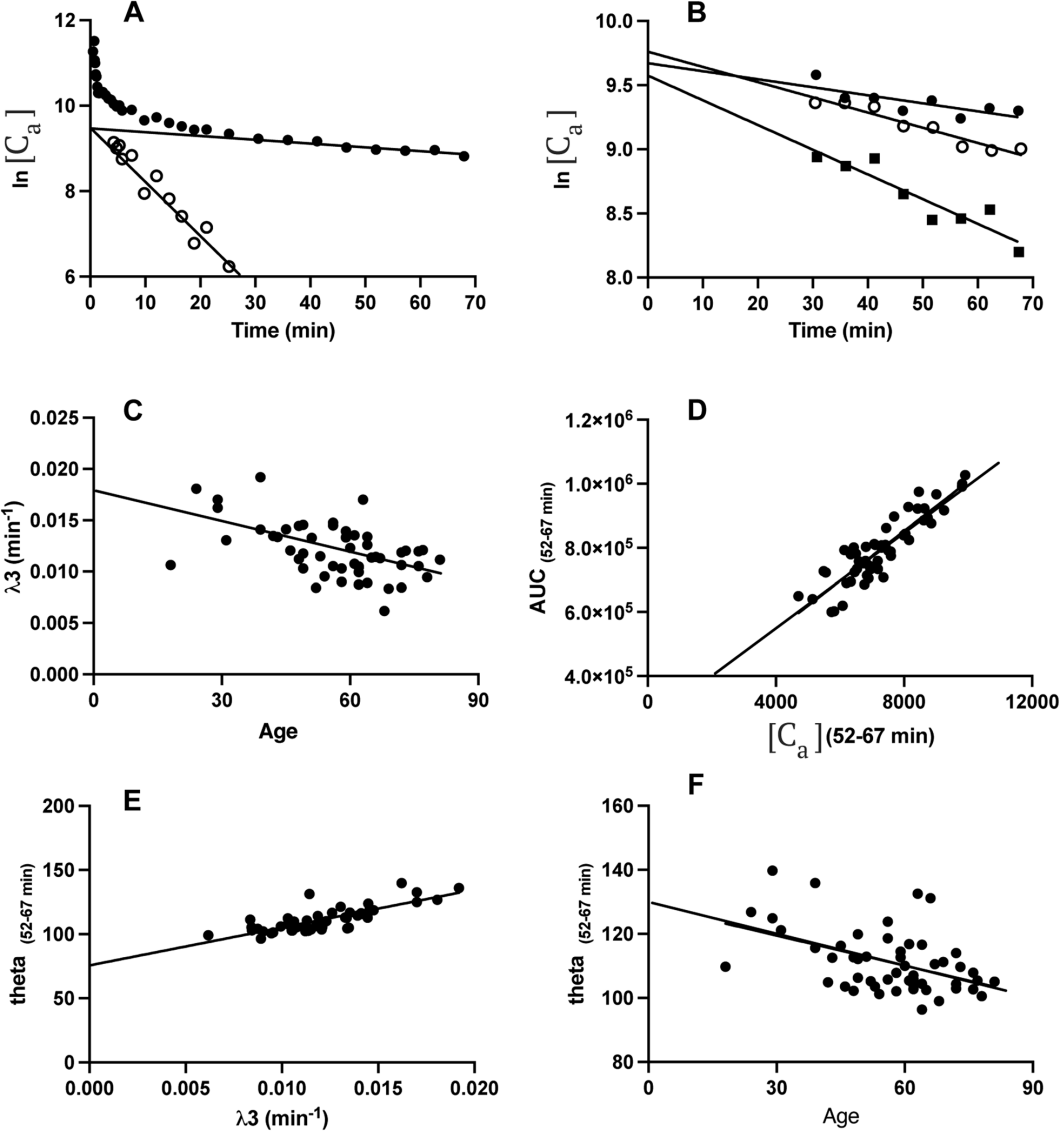
(**A**) Scatter plot of the natural logarithm of the arterial FDG concentration as a function of circulation time from a representative subject. Here the measured IDIF is decomposed into its late component (λ3 and its ordinate intercept C) and the intermediate component (λ2 and its ordinate intercept, B), obtained by the method of residuals of the Cλ3 phase. The open circles indicate the residuals of the partial decomposition of Cλ3. In this example, the ordinate intercepts of the linear regressions corresponding to B and C are nearly identical. For clarity of presentation; we omit the corresponding decomposition of the fastest phase, Aλ1. (**B**) Semi-logarithmic plots of the clearance phase from representative slow, intermediate and fast eliminators. (**C**) The relationship between λ3 and subject age from (*n* = 52) patients scanned in Aarhus. (**D**) The corresponding empirical relationship between the total measured AUC_(0–67 min)_ and the mean arterial concentration measured during the final three frames of the PET recording (Ca_(52–67 min)_. The empirical relationships between the normalized arterial integral measured during the final three frames (theta_(52–67 min)_ as functions of (**E**) the individual late phase FDG clearance rate constant (λ3) and (**F**) age of the subjects

In Aarhus and Bern we noted the time to first sign of blood radioactivity and the time to peak radioactivity in each case. We then plotted the natural logarithm of the blood radioactivity as a function of circulation time. Linear regression of the late phase semi-log plots gave the plasma radioactivity concentration as Ce^−λ3(t)^, where C is a constant and λ3 is the fractional rate constant for the renal clearance of plasma FDG. Next, the extrapolate magnitude of Ce^−λ3(t)^ was subtracted from the total measured IDIF concentration series, and the intermediate phase calculated as Be^−λ2(t)^ (Fig. 1A), followed by an additional decomposition of the residuals to isolated the early, rapid phase of FDG distribution, defined as Ae^−λ1(t)^. Thus, the entire IDIF was described using a function similar to the exponential form used previously by Feng et al. [29], namely,

$${\text{C}}_{{\text{a}}} \left( {\text{t}} \right) \, = {\text{ Ae}}^{{ - \lambda {1}\left( {\text{t}} \right)}} + {\text{Be}}^{{ - \lambda {2}\left( {\text{t}} \right)}} + {\text{ Ce}}^{{ - \lambda {3}\left( {\text{t}} \right)}}$$

For each individual, we calculated the mean plasma FDG concentration measured during the final 15 min of the PET recording (Ca_(52–67 min)_), the measured area under the curve (AUC) or plasma integral to the end of the PET recording, and the corresponding AUCs estimated from one, two, and three exponential terms. The measured area under the curve (AUC) to 67 min (AUC_(0–67 min)_) was calculated for each case, along with the corresponding AUCs calculated for the mono-, bi, and tri-exponential functions, which were then calculated as percentage recovery of the measured AUC, with evaluation of the exponential components beginning at the time (circa 0.5 min) of the first appearance of radioactivity in the measured IDIF. The final arterial FDG concentration was calculated as the mean of three measurements during the last three frames (52–67 min), and the corresponding normalized arterial input (theta, min) was the ratio of the AUC_(0–67 min)_) to the measured Ca_(52–67 min)_.

The relationships between the various plasma kinetic parameters and age of the individual were calculated as a correlation matrix, separately for Aarhus (*n* = 52) and Bern (*n* = 24) data. An initial exploration of the correlations revealed a number of relationships useful for modeling of the IDIF and calculating the complete AUC from various late phase recordings, including a single frame PET recording corresponding to 52–67 min post injection. The magnitude of the standard net blood–brain clearance (*K*_i_; ml g^−1^ min^−1^) in WM and GM was calculated by linear graphic analysis of PET frames in the interval 12–67 min post injection relative to the complete measured IDIFs, using the mean ordinate intercept for GM (*V*_*D*_, *K*_1_/*k*_*2*_) as one point in the voxelwise two-point Patlak analysis of the magnitude of FDG-*K*_i_, separately for whole GM and whole WM.

We performed four kinds of single-point Patlak analysis, using pharmacokinetic relationships that emerged from the IDIF analyses, as described below. In Method 1, we defined the limiting slope of the semi-log transformation of the FDG blood concentration during the interval 35–67 min, and calculated λ3 (min^−1^), the fractional rate constant for the elimination of FDG from arterial blood. We then obtained a population mean correction factor for the AUC of the Ce^−λ3(t)^ term for each individual to estimate the AUC of their complete IDIF, and thereby calculated for each individual the terminal theta_(52–67 min)_ relative to the terminal blood concentration (Ca_(52–67 min)_. We then estimated *K*_i_ as a two point Patlak-Gjedde plot defined by the ordinate intercept in the IDIF evaluation for GM (*V*_*D*_; 0.55 ml g^−1^ at both scanning sites) and the tracer distribution volume calculated from the mean radioactivity concentrations measured in GM, WM, and blood during the final three frames of the PET recording. Method 2: From the site-specific empirical relationships between AUC_(0–67)_ as a function of Ca_(52–67 min)_, we similarly calculated *K*_i_ from Ca_(55–67 min)_ as a two point Patlak plot. Method 3: We similarly used the site-specific empirical relationships between theta_(52–67 min)_ and λ3 measured during the interval 35–67 min post injection. Method 4: We similarly used the site-specific empirical relationships between theta_(52–67 min)_ and subject age in the two populations.

## Results

There were 52 cases from Aarhus of mean (SD) age 56 (14) years. The mean (SD) time to first plasma radioactivity was 0.24 (0.05) min and the mean (SD) time to peak radioactivity was 0.56 (0.09) min. There were 24 cases from Bern of mean (SD) age 60 (16) years. The mean (SD) time to first plasma radioactivity was 0.51 (0.11) min and the mean (SD) time to peak radioactivity was 0.80 (0.11) min.

A semi-logarithmic plot of the FDG-IDIF as a function of time shows the intermediate (λ2) and slow (λ3) phases of the elimination of FDG in a representative individual, with omission of the decomposition of the fast (λ1) phase for the sake of clarity (Fig. 1A), and the estimation of λ3 for individuals with slow, average, and rapid eliminations (Fig. 1B). In the correlation matrix of pharmacokinetic terms, there were very high correlations between the terms of each component of the tri-exponential function, i.e., A and λ1, B and λ2, and C and λ3, which held equally for Aarhus and Bern data (Table 1). Indeed, these relationships are trivial, since a high fractional rate constant for a process will necessarily extrapolate to a high concentration. The magnitudes of A and B correlated positively, as did the magnitudes of λ1 and λ2 (significantly so in the Aarhus data). In both data sets, there were high correlations between the magnitudes of the late phase intercept C and the measured AUC_(0–67 min)_, as well as with the corresponding late phase plasma FDG concentration (Ca_(52–67 min_). The magnitude of the late phase elimination rate constant λ3 correlated negatively with the magnitude of Ca_(52–67 min)_ in Aarhus data, positively with theta_(52–67 min)_ in both data sets, and inversely with age in the Aarhus data set.

**Table 1:** Matrix of correlations between various FDG pharmacokinetic parameters

	A	λ1	B	λ2	C	λ3	AUC_(0–67 min)_	Ca_(52–67 min)_	theta_(52–67 min)_	Age
A	1	+ 0.514** + 0.684**	+ 0.555*** + 0.406	+ 0.448** + 0.147	+ 0.006 + 0.320	− 0.115 − 0.012	+ 0.180 + 0.463*	+ 0.141 + 0.508*	− 0.075 + 0.007	+ 0.284* + 0.373
λ1		1	+ 0.284 + 0.573**	+ 0.419** + 0.237	+ 0.156 + 0.192	+ 0.096 − 0.043	+ 0.019 + 0.322	− 0.148 + 0.375	+ 0.159 + 0.057	+ 0.130 + 0.508*
B			1	+ 0.503*** + 0.577*	− 0.131 + 0.159	− 0.376* – 0.096	+ 0.054 + 0.439	+ 0.241 + 0.420	− 0.187 + 0.109	+ 0.329* + 0.305
λ2				1	− 0.152 − 0.130	− 0.022 + 0.005	+ 0.032 − 0.024	− 0.157 − 0.157	− 0.127 + 0.222	+ 0.176 + 0.290
C					1	+ 0.514*** + 0.737***	+ 0.510*** **+ 0.874#**	+ 0.552*** + 0.638*	+ 0.156 + 0.548*	− 0.180 + 0.266
λ3						1	− 0.171 + 0.379	− 0.436** − 0.033	**+ 0.798#** **+ 0.846#**	0.514*** + 0.084
AUC_0–67 min)_							1	**+ 0.678#** **+ 0.885#**	− 0.270 + 0.325	+ 0.145 + 0.324
Ca_(52–67 min)_								1	− 0.564*** − 0.122	+ 0.244 + 0.315
theta_(52–67 min)_									1	− 0.510*** + 0.168
Age										**1**

A, B, and C (arbitrary plasma concentration units) and λ1, λ2 and λ3 (fractional rate constants; min^−1^) refer to the fitting of the tri-exponential function describing the declining blood FDG concentrations measured during 67 min. AUC_(0–67 min)_ refers to the integration of the measured blood curve to the end of the PET recordings (in arbitrary concentration units). Ca_(52–67 min)_ is the mean blood concentration of FDG measured during the final three frames of the PET recording. Theta_(52–67 min)_ is their ratio (AUC_(0–67 min)_/Ca_(52–67 min)_), also known as the normalized arterial input, prevailing during the final three frames of the PET recording. Age is the patient age in years. We calculated the correlations coefficients separately for groups of 52 subjects (Aarhus; upper row) and 24 subjects (Bern; lower row), where bold font indicates uncorrected significant correlation coefficients: *P < 0.05, **P < 0.002. Only ***P < 0.0002 #*P* < 0.00001 would survive correction for (*n* = 45 or 24) correlations

The population mean pharmacokinetic results for Aarhus and Bern are presented in Table 2. The magnitude of λ3 declined significantly with age in the Aarhus data (Table 2**, **Fig. 1C), but not so in the Bern data. Relative to the complete IDIF, the population mean estimates of FDG-*K*_i_ from the Aarhus data were 0.0350 ± 0.0086 ml g^−1^ min^−1^ for GM and 0.0143 ± 0.0099 ml g^−1^ min^−1^ for WM, with practically identical results for the Bern data (Table 3). Similarly, the ordinate intercepts in the GM linear graphic analysis (V_D_; 0.55 ml g^−1^) were identical in the two data sets (Table 3), which therefore constituted the initial point in the two-point linear graphic analyses used in Methods 1–4.

**Table 2:** Population based results from the pharmacokinetic analysis of FDG in arterial blood and (*n*=24) patients from Bern

	Aarhus (*n* = 52)	Bern (*n* = 24)
AUC_(0–67),_ measured	100.0 ± 13.1	100.0 ± 12.3
Ce^−λ3(t)^ (Method 1)	84.7 ± 3.9	83.6 ± 2.5
Be^−λ2(t)^ + Ce^−λ3(t)^	95.5 ± 2.1	94.5 ± 1.6
Ae^−λ1(t)^ + Be^−λ2(t)^ + Ce^−λ3(t)^	98.7 ± 2.4	97.8 ± 2.1
Age (years)	56.5 ± 14.1	59.7 ± 15.2
λ3 (min^−1^)	0.0121 ± 0.0026	0.0094 ± 0.0019
theta_(52–67 min)_ (min)	111.3 ± 9.8	100.8 ± 6.7
λ3 (min^−1^) versus age (years) (see Fig. 1C)	λ3 = − 0.0000936*(age) + 0.0174 (*r* = − 0.514; p > 0.0001)	λ3 = 0(age) + 0.00946 (*r* = − 0.011; not significant)
AUC_(0–67 min)_ versus Ca_(52–67)_ (see Fig. 1D, Method 2)	AUC_(0–67 min)_ = 63.2*(Ca_(52–67 min)_) + 374 (*r* = + 0.678; *p* < 0.00001)	AUC_(0–67 min)_ = 104.0(Ca_(52–67)_) + 2.9 (*r* = + 0.885; *p* < 0.00001
theta_(52–67 min)_ (min) versus λ3 (min^−1^) (see Fig. 1E, Method 3)	theta_(52–67 min)_ = 2953*(λ3) + 75.57 (*r* = + 0.796; *p* < 0.00001)	theta_(52–67 min)_ = 2971(λ3) + 72.89 (*r* = + 0.846; *p* < 0.00001)
theta_(52–67 min)_ (min) versus age (years) (see Fig. 1F, Method 4)	theta_(52–67 min)_ = − 0.313*(age) + 129 (*r* = − 0.46; *p* < 0.0002)	theta_(52–67 min)_ = 0.0477(age) + 98.0 (*r* = + 0.17, n.s.)

We obtained population-based results from the pharmacokinetics of FDG in arterial blood measured during PET recordings lasting 67 min, based on observations in (*n* = 52) patients from Aarhus and (*n* = 24) patients in Bern. The total measured AUC_(0–67)_ was calculated from the IDIF measured during the complete dynamic recordings, and normalized to a mean of 100 arbitrary units of concentration-min. This AUC_(0–67)_ is shown along with the percentage AUC recoveries from (1) the final phase of the tri-exponential function (ACe^−λ3(t)^), (2) the sum of the intermediate and final phases (Be^−λ2(t)^ + Ce^−λ3(t)^), and (3) the complete tri-exponential function (Ae^−λ1(t)^ + Be^−λ2(t)^ + Ce^−λ3(t)^). We also report some relationships discovered for one or both sites, i.e., (1) the magnitude of λ3, which corresponds to renal clearance of FDG, versus subject age, (2) theta_(55–67 min)_ versus λ3, AUC_(0–67 min)_ versus Ca_(52–67 min)_, and theta_(52–67 min)_ versus subject age. Methods 1,2,3, and 4 refer to the specified empirical relationships for estimating the magnitude of FDG-*K*_i_ in brain from a two-point Patlak linear graphic analysis defined by (1) the population mean gray matter V_D_ (0.55 ml g^−1^) and (2) estimations of AUC_(0–67 min)_/ theta_(52–67 min)_ obtained from late phase recordings. In Method 1, we estimated the AUC_(0–67)_ from the individual Ce^−λ3(t)^scaled by the population mean percentage AUC recoveries (84.7% in Aarhus, 83.6% in Bern). In Method 2, we estimated AUC_(0–67)_ and thence theta_(52–67 min)_ from the individual Ca_(52–67 min)_ and the site-dependent empirical relationship. In Method 3, we estimated theta_(52–67 min)_ from the individual λ3 based on the final 30 min of the recording and the site-dependent empirical relationship. In Method 4, we estimated theta_(52–67 min)_ from the individual subject age and the site-dependent empirical relationship

**Table 3:** The population mean (SD) values for net influx of FDG (*K*_i_; ml g^−1^ min^−1^) from blood to brain by standard IDIF method and four simplified methods

	Aarhus (*n* = 52)		Bern (*n* = 24)	
	GM	WM	GM	WM
Standard IDIF *K*_i_ (ml g^−1^ min^−1^) V_D_ (ml g^−1^)	0.0350 ± 0.0086 0.55 ± 0.19	0.0143 ± 0.0099 0.51 ± 0.13	0.0345 ± 0.0091 0.55 ± 0.15	0.0153 ± 0.0047 0.45 ± 0.10
Method 1 *K*_i_ (ml g^−1^ min^−1^) %error	0.0336 ± 0.0077 − 3.5 ± 7.2% *r* = 0.93**	0.0119 ± 0.0026 − 16.7 ± 20.2% *r* = 0.44**	0.0354 ± 0.0099 + 2.5 ± 11.3% *r* = 0.86**	0.0135 ± 0.0040 − 11.6 ± 13.1% *r* = 0.94**
Method 2 *K*_i_ (ml g^−1^ min^−1^) %error	0.0338 ± 0.0090 − 3.7 ± 7.2% *r* = 0.71**	0.0119 ± 0.0031 − 16.7 ± 19.2% *r* = 0.58**	0.0333 ± 0.0093 − 3.4 ± 10.6% *r* = 0.86**	0.0127 ± 0.0037 − 16.7 ± 12.3% *r* = 0.94**
Method 3 *K*_i_ (ml g^−1^ min^−1^) %error	0.0352 ± 0.0118 + 0.7 ± 25.1% *r* = 0.61**	0.0123 ± 0.0044 − 13.9 ± 31% *r* = 0.33*	0.0346 ± 0.0098 + 0.4 ± 12% *r* = 0.88**	0.0132 ± 0.0040 − 13.6 ± 11.7% *r* = 0.97**
Method 4 *K*_i_ (ml g^−1^ min^−1^) %error	0.0348 ± 0.0090 − 0.7 ± 7.3% *r* = 0.68**	0.0124 ± 0.0032 − 13.2 ± 8.1% *r* = 0.36*	0.0346 ± 0.0098 + 0.2 ± 11.4% *r* = 0.86**	0.0132 ± 0.0039 − 13.6 ± 12.9% *r* = 0.94**

We present population mean (SD) values for net influx of FDG (*K*_i_; ml g^−1^ min^−1^) from blood to the entire gray matter (GM) and white matter (WM) using data from Aarhus (*n* = 52) and Bern (*n* = 24), along with the error relative to the complete IDIF results, and the corresponding ordinate intercepts (V_D_; ml g^−1^). We tested four simplified methods based on pharmacokinetic findings from Table 2 with respect to their mean *K*_i_ values, the percentage error relative to standard IDIF values, calculated as [(Method-IDIF)/IDIF], where the IDIF method entails linear graphic analysis relative to the brain radioactivity concentrations measured during the interval 12–67 min. In all four simplified methods, the mean GM V_D_ estimate from the IDIF regression (0.55 ml g^−1^) served to constrain the estimation of *K*_i_. Method 1: From semi-logarithmic analysis of the FDG blood concentration during the interval 35–67 min, we calculated λ3 (min^−1^), the fractional rate constant for the elimination of FDG from arterial blood. The AUC extrapolated to the time of first appearance of the radioactivity peak corresponded to a mean of 85% of the total measured AUC (Aarhus) or 84% (Bern). Using these correction factors, we estimated the complete AUC, and calculated the terminal theta_(52–67 min)_ relative to the terminal blood concentration (Ca_(52–67 min)_. We then estimated *K*_i_ as a two point Patlak plot defined by gold standard population ordinate intercept (V_D_; ml g^−1^) and the tracer distribution volume calculated from the mean radioactivity concentrations measured in GM, WM, and blood during the final three frames (52–67 min) of the PET recording. Method 2: From the site-specific empirical relationships between AUC_(0–67)_ as a function of Ca_(52–67 min)_, we similarly calculated *K*_i_ from Ca_(52–67 min)_ as a two point Patlak plot. Method 3: We similarly used the site-specific empirical relationships between theta_(52–67 min)_ and λ3 measured during the interval 35–67 min post injection. Method 4: We similarly used the site-specific empirical relationships between theta_(52–67 min)_ and subject age in the two populations. Significance of Pearson correlation coefficients: (*) *p* < 0.05; (**) *p* < 0.001

The percentages of the total AUC recovered from the late phase component of the IDIF (Ce^−λ3(t)^) showed excellent agreement between Aarhus (85%) and Bern (84%) (Table 2); these recoveries served in Method 1 to correct the partial IDIF to the complete IDIF-AUC for each individual, from which we then calculated the magnitude of theta in the interval 52–67 min post injection, knowing the measured Ca_(52–67 min)_. At both research sites, Method 1 gave good agreement with the corresponding complete IDIF estimates of FDG-*K*_i_ for GM and WM.

The relationship between AUC_(0–67)_ as a function of Ca_(52–67 min)_ for the Aarhus data set (Fig. 1D), and the corresponding regression equations for both sites (Table 2) provide the basis for Method 2, where the missing AUC_(0–67 min)_ is estimated from the individual Ca_(52–67 min)_. Method 2 estimates of FDG-*K*_i_ using the site-specific empirical relationships were in good agreement with IDIF results (Table 3).

The relationship between theta_(52–67 min)_ (min) versus individual λ3 (min^−1^) for the Aarhus data set (Fig. 1E) and the corresponding regression equations for both sites (Table 2) provide the basis for Method 3, where the unknown theta_(52–67 min)_ is estimated from the individual λ3, which was calculated from the semi-logarithmic plot of the plasma FDG concentration as a function of time during the interval 30–67 min post injection. Method 3 estimates of FDG-*K*_i_ using the site-specific empirical relationship were in good agreement with IDIF results (Table 3).

The relationship between theta_(52–67 min)_ (min) versus age from the Aarhus data set (Fig. 1F) was significant, but not so for Bern data (Table 2). The site specific empirical relationships provided the basis for Method 4, where the unknown theta_(52–67 min)_ is estimated from the individual subject’s age, thus affording estimation of the FDG-*K*_i_ from a single PET frame measured during 52–67 min post injection. Method 4 estimates of FDG-*K*_i_ from both sites were in good agreement with IDIF results (Table 3), despite the lack of age-dependence in the Bern data.

## Discussion

Our motivation in conducting these studies derives from the logistic difficulty and expense of conducting dynamic PET recordings for the quantitation of FDG net influx (*K*_i_) and CMR_glc_. Recent developments in IDIF-based methods have given new impetus to quantitative FDG-PET, which may avoid certain interpretative pitfalls related to global normalization or other semi-quantitative approaches. Such approaches include interrupted PET recordings consisting of early and late phase sequences [30], or deep learning-based procedure for conversion of a single static frame to a parametric FDG-*K*_i_ image [31]. Indeed, we have recently show that a two-phase, interrupted PET sequence gives excellent agreement with the FDG-*K*_i_ evaluation in tumors from the present group of oncology patients examined with the Biograph Vision Quadra [32]. Now focusing on brain uptake, we herein tested a number of procedures designed to evaluate FDG-*K*_i_ from relatively brief dynamic or static late-phase recordings, which are based on simple pharmacokinetic principles applied to an IDIF from the descending aorta, as acquired through bed motion (Aarhus), or from simultaneous recording of vascular and cerebral structures (Bern). While complete methodological harmonization between sites was impossible due to the differing instrumentation, the scanners at Bern and Aarhus have similar spatial resolution and TOF resolution. However, the ten-fold higher sensitivity of the LAFOV scanner in Bern [33] lead us to make reconstructions of the Bern data in shorter frames. We rather see these methodological differences as a strength of the present study, insofar as we establish the generalizability of our methods for single frame quantitation of FDG-*K*_i_.

There are a number of advanced approaches for noninvasive (blood-free) quantitation of FDG kinetics, or quantitation from abbreviated scanning protocols. IDIFs from the internal carotid as defined in MRI gave good agreement with blood measurements during 60 min dynamic PET recordings [34]. Recent work with long axial field-of-view PET scanners showed that shortened protocols, which might involve double scanning or double administration of FDG, might be used to get accurate estimates microparameters, i.e., the unidirectional blood–brain clearance (*K*_1_) as well as net tracer influx (*K*_i_) [30, 32]. In other studies, population-based input functions were calculated by normalization of an IDIF measured during the interval 30–60 min post-injection gave negligible error in the mean estimation of FDG-*K*_i_ [23]. We and others similarly reported good agreement between a population-based aorta IDIF scaled to a brief recording interval and a complete IDIF measured during 60 min with the Biograph Vision Quadra PET/CT [22, 24]. Alternately, the data-driven Source to Target Rotating Estimation (STARE) approach calculates the two-tissue compartment microparameters (K_1_,k_2_,k_3_) for FDG by formulating the TAC in a target region as a function of a “source region,” thus making superfluous the measurement of blood concentration [35]; that approach gave good agreement (5% bias) with IDIF results.

The representation of the arterial input curve as a multi-exponential function dates to among the earliest reports of FDG-PET in humans [1]; indeed, this is a general property of many radiotracers for PET following bolus intravenous administration [36]. The three phases of the arterial FDG curve in the present analysis, which are very similar to those in the formulation by Feng et al. ([23, 29], might be attributed to rapid mixing of the bolus in the blood pool (Ae^−λ1(t)^), partitioning of plasma FDG into bodily tissues (Be^−λ2(t)^), and renal elimination of FDG from the body (Ce^−λ3(t)^). In both data sets, the correlations among the various parameters of the tri-exponential function (Table 1) showed considerable covariance of canonical pairs of parameters (for example A and λ1), which is to be expected. Mainly in the Aarhus data, patient age correlated certain kinetic parameters, which might reflect age-dependent changes in hemodynamics, hematocrit, or some other factor governing dynamics of the distribution of the bolus in bodily tissues. We have earlier shown that the magnitude of λ3 for the dopamine synthesis tracer [^18^F]fluorodopa declines with age in healthy subjects, which we attributed to declining renal function [37]. In the Aarhus data set, we see a robust recapitulation of this phenomenon for FDG, with the implication that the fractional rate constant of renal elimination of FDG declines by half across the human life span. Insofar as the late phase of the empirical tri-exponential function proved to be a major determinant of the total AUC for FDG, it might follow that dosimetry requirements should decline with age; in effect the bioavailability of a given FDG dose increases with age. However, this key correlation of λ3 with age was not present in the Bern data set, which may reflect the smaller sample size, and factors related to the performance of the particular instrumentation in extracting the tail of the IDIF. Pharmacokinetic analysis of data from further sites could better establish the generalizability of the age-dependencies seen in the Aarhus IDIF data.

Returning to the importance of the late phase governed by λ3 in determining the total AUC, we see that Ce^−λ3(t)^ extrapolated to the time of injection accounted for 85% (Aarhus) or 84% (Bern) of the total AUC from time of injection to the end of the 67 min recording. In Method 1, using these recoveries as correction factors, ln-transformation of a dynamic data recorded during the interval 35–67 min post injection gives a robust estimation of the total AUC (Table 2**)**, with relatively little error due to uncertainty in the contributions of the early pharmacokinetic phases, namely Ae^−λ1(t)^ + Be^−λ2(t)^. Thus, Method 1 gave good estimates of GM *K*_i_ in both data sets, with a tendency for 10% underestimation in the evaluation of WM *K*_i_. As such, the initial 35 min of the recording seems hardly necessary for quantitation of net brain uptake in whole GM, so long as the late phase data are sufficient for a good estimation of Ce^−λ3(t)^ in the blood compartment. We note that this application of a two-point linear graphic analysis is constrained by knowledge of the ordinate intercept in the GM linear graphic analysis (0.55 ml g^−1^), which corresponds well to the ratio K_1_/k_2_ (V_D_) from compartmental analysis of human FDG-PET data [1–3].

In both data sets, we found strong relationships between AUC_(0–67 min)_ and the mean arterial concentration measured during the final three frames of the PET recording (Ca_(52–67 min)_) (Fig. 1D). Put another way, the mean blood pool concentration measured during the final 15 min of the recording bears the history of the preceding pharmacokinetic processes, such that a measurement of Ca_(52–67 min)_ enables the estimation of the complete AUC_(0–67 min)_. Therefore, Method 2 gives FDG-*K*_*i*_ estimates that are in good agreement with the corresponding IDIF estimates (Table 3). Similarly, in Method 3, the strong relationship between theta_(52–67 min)_ and λ3 gave equally good FDG-*K*_i_ results relative to the full dynamic IDIF method (Table 3). In Method 4, the site-specific empirical relationships between theta_(52–67 min)_ and subject age also performed similarly well (Table 3), despite the lack of significant age-dependence in the Bern data set (Table 2). Overall, Methods 1 and 3, which required serial measurements of the arterial FDG concentration during 35–67 min post injection, performed similarly for the calculation of FDG-*K*_i_ in GM as did Methods 2 and 4, which entailed brain and blood pool measurements during a much briefer interval, namely 52–67 min post injection. The relatively higher errors in WM seen for all of the two-point linear graphic analysis methods may reflect our use of a GM estimate of V_D_, which slightly exceeds that for WM. Overall, we see good agreement between Aarhus and Bern FDG-*K*_i_ results, despite the imperfect harmonization of data acquisition procedures. We cannot presently account for the lacking age-dependence of theta_(52–67 min)_ and λ3 in the Bern data, especially considering the good accord between sites for other relationships presented in Table 2.

In our earlier analysis of the Bern data, a dynamic recording covering the first 10–15 min post-injection, followed by a brief scan an hour later, gave excellent quantitation of FDG trapping in tumor lesions, again relative to an IDIF captured by the long axial FOV Biograph Vision Quadra tomograph [32]; we are currently investigating an interrupted scanning approach for the quantitation of FDG uptake in brain. Naturally, recording a single dynamic sequence is logistically easier than obtaining early and late-phase recordings. In the present study we discovered a set of simple pharmacokinetic characteristics of the FDG arterial input curve, namely the strong inter-relationships between individual magnitudes of total AUC, the normalized input (theta), and arterial tracer concentration at the end of the recording. As such the complete AUC of the IDIF can be estimated from relatively brief late phase blood pool measurements.

## Conclusions

We show that single static frames recorded at approximately one hour after FDG administration suffice for accurate quantitation of the net tracer influx (*K*_i_) in brain, relative to linear graphic analysis of the entire dynamic sequence. This reflects certain canonical relationships between the mean FDG concentrations measured in the descending aorta at one hour after administration and the total AUC of the IDIF to the end of the recording; in effect, the various simplified methods presented herein are functionally equivalent. Notably, the generalizability of present methods remains uncertain for patients with diabetes [10], renal failure, pharmacological treatments, or other conditions potentially influencing FDG plasma kinetics. However, present results add to the repertoire of methods for “bloodless” quantitation of FDG-PET from relative brief recordings.

## Data Availability

Primary data are available upon reasonable request. No participants were aged less than 18 years.
